# Abdominal cocoon syndrome, a diagnostic challenge affecting the treatment. A rare case report from Somalia

**DOI:** 10.1093/jscr/rjad107

**Published:** 2023-03-21

**Authors:** Schauki Mahmoud, Abdurrahman Hashi Salad, Aisha Abdirahman Abdullahi

**Affiliations:** General Surgery Department, Kalkaal Hospital, Mogadishu, Somalia; General Surgery Department, Kalkaal Hospital, Mogadishu, Somalia; General Surgery Department, Kalkaal Hospital, Mogadishu, Somalia

**Keywords:** abdominal cocoon syndrome, intestinal obstruction, computed tomography, sclerosing encapsulated peritonitis

## Abstract

Abdominal cocoon syndrome is defined as idiopathic encapsulation of the bowel within a fibrocollagenous membrane and is considered as a rare cause of small bowel obstruction. A 15-year-old female presented complaining of right lower abdominal pain, distension and vomiting for 24 hours with previous similar attacks in the last four years. She had no another significant medical or surgical history. Computed tomography study revealed matted mildly distended bowel loops centrally with a suspicion of acute appendicitis. Next day, abdominal pain was the only clinical finding and acute appendicitis was the primary diagnosis. During surgery, most of the small bowel was found to be encapsulated within a cocoon-like fibrous membrane. The appendix was congested. Appendectomy, full resection of the membrane and dense adhesiolysis were performed. Herein, we will present the first reported case from Somalia and discuss the radiological findings affecting the management for such a rare disease.

## INTRODUCTION

Abdominal cocoon syndrome (ACS) is a rare disease that is defined as idiopathic partial or total encapsulation of the bowel within a fibrocollagenous membrane and is considered as a rare cause of small bowel obstruction (SBO) [1].

Peritoneal encapsulation (PE) is a congenital abnormality, in which part or all of the small intestine is covered with a thin peritoneal membrane and without intestinal adhesions and rarely characterises by intestinal obstruction [2, 3].

Sclerosing encapsulated peritonitis (SEP) is an acquired disease including two types: iatrogenic SEP or ACS and secondary SEP. Secondary SEP can be found in patients with peritoneal dialysis, tuberculosis, ventriculoperitoneal shunt, povidone peritoneal lavage, etc. [2, 4].

The histopathology study for the membrane of ACS is characterised by the presence of a fibrocollagenous membrane with an inflammatory process, which is considered the corner stone to differentiate SEP and PE [2].

Herein, we will present the first reported case of ACS from Somalia and discuss the radiological findings affecting the management for such a rare disease.

## CASE PRESENTATION

A 15-year-old female was admitted to the hospital complaining of right lower abdominal pain and vomiting for 24 hours. The pain started as severe in the periumbilical area and the right iliac fossa (RIF) and associated with vomiting, nausea and constipation. She had previous similar attacks in the last 4 years with average one attack per 3 months, which resolved within hours with anti-pain medication. She had no other significant medical or surgical history. The patient was referred to us from another hospital with a diagnosis of acute appendicitis. On examination, she was moderately ill looking, stable, afebrile and with regurgitation. Abdominal exam revealed mild distension centrally with tenderness on RIF and periumbilical area. Abdomen US revealed dilated cecum with reduced bowel movement and the appendix could not be visualised. Abdominal enhanced computed tomography (ECT) revealed matted small bowel in the center of the abdomen with mild proximal small bowel distension and minimal free fluid in the pelvis, mostly due to acute appendicitis. Laboratory investigations were within normal limits with mildly elevated CRP = 7.4 mg/L.

**Figure 1:**
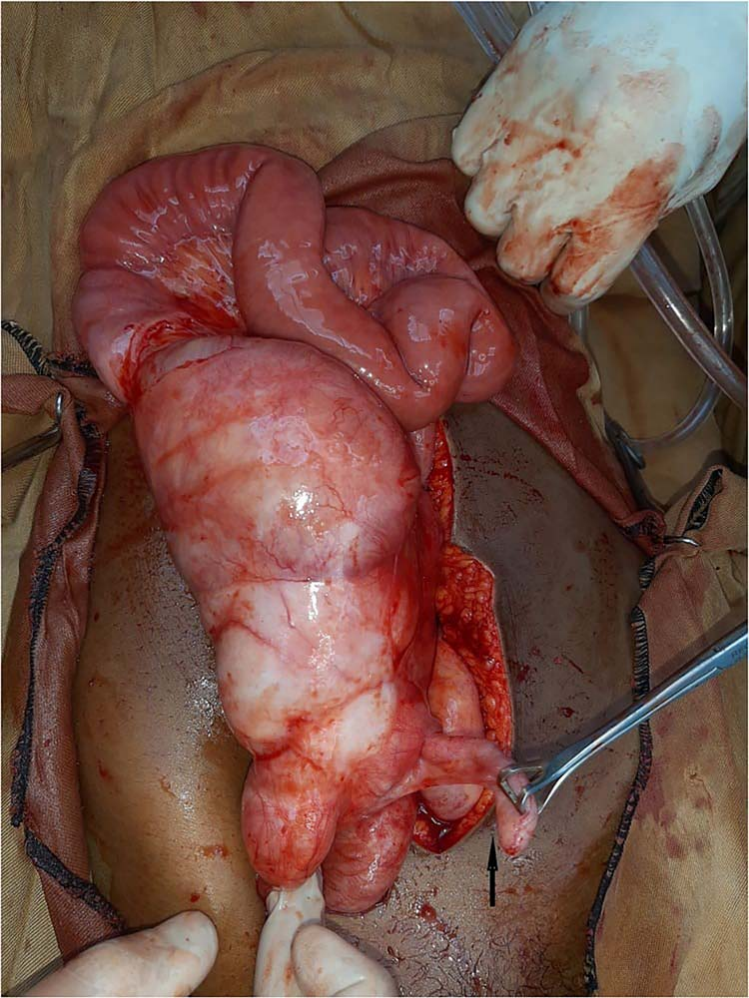
Intra-operative view: most of the small bowel was encapsulated within thickened white, fibrous cocoon like membrane including the distal half of jejunum, ileum and cecum. The appendix was not included within the membrane and congested (black arrow).

**Figure 2:**
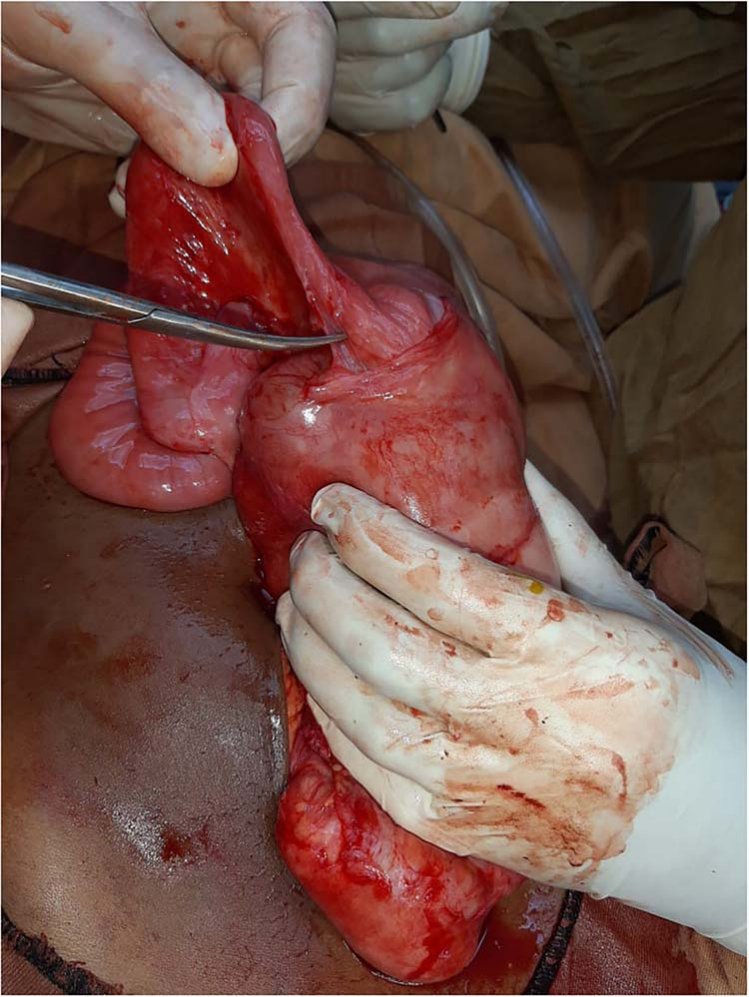
Intra-operative view: starting sharp dissection for the membrane and the small bowel dense adhesions.

**Figure 3:**
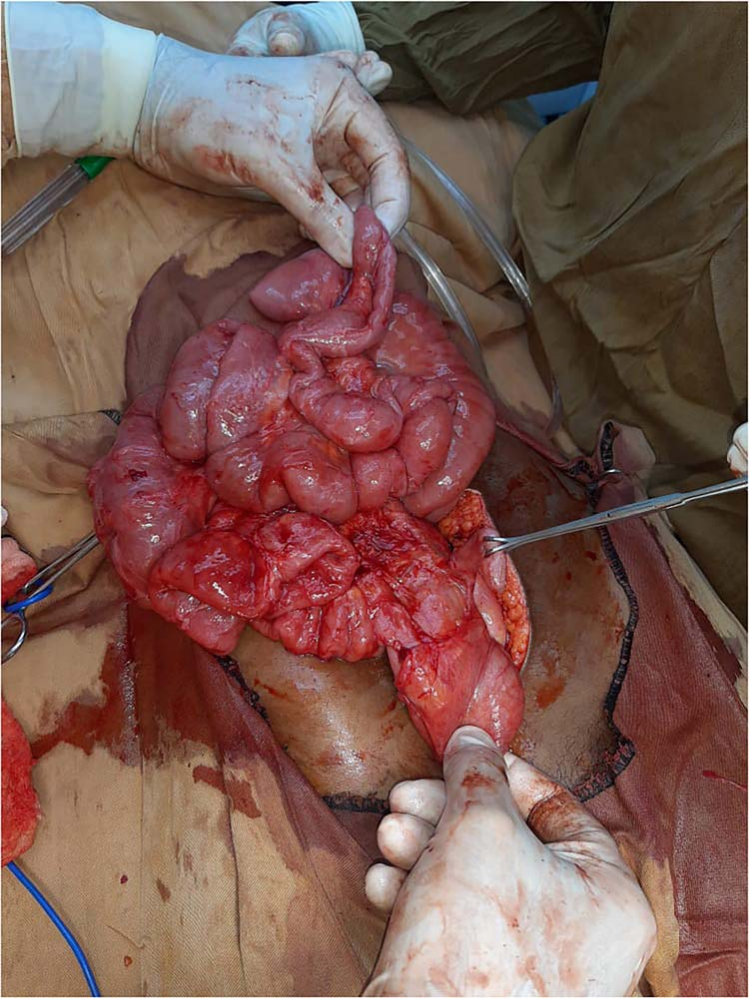
Intra-operative view: complete membrane resection with full small intestine adhesiolysis.

Then, the patient was scheduled for exploratory laparotomy. Due to a financial issue, laparotomy was postponed to the next day. When the patient was examined again, she was improved with neither distended abdomen nor vomiting and passed flatus. The pain was localised on RIF with marked tenderness. Acute appendicitis was the primary diagnosis. After intubation, a movable mass in the center of the abdomen was palpated and midline incision was carried out. There was a mild amount of serous fluid in the pelvis and most of the small bowel was encapsulated within a thickened white, cocoon-like fibrous membrane, including the distal half of the jejunum, ileum and cecum. The appendix was not included within the membrane and was congested (Fig. 1). A careful sharp dissection and excision of the dense membrane with lysis of the severe adhesions between the bowel loops were performed (Figs 2 and 3). The small intestine was viable without any injury during dissection. Appendectomy was performed. No malrotation of the bowel was found.

The post-operative course was uneventful and she was discharge on post- operative day 4.

The histopathology study revealed a fibrocollagenous membrane with foci of moderate active chronic inflammation, early appendicitis with mild periappendicitis.

## DISCUSSION

The symptoms of ACS are non-specific as recurrent abdominal distension, nausea, vomiting, abdominal pain and a palpable mass [1, 5]. Most patients suffer from recurrent attacks of subacute SBO that relief spontaneously. In our patient, the attack of SBO subsided in 24 hours and the indication of our surgery was the abdominal pain that was misdiagnosed radiologically and clinically as acute appendicitis. The histopathology study revealed mild periappendicitis. This fact raises the question about the necessity of our surgery if the real diagnosis was established by ECT pre-operatively.

Due to the rarity of ACS and its non-specific symptoms, ACS is commonly misdiagnosed. Recently, ECT is the modality of choice to detect the diagnosis of ACS. Identifying a fibrous membrane surrounding the small intestine is considered the specific radiological finding [4].

**Figure 4:**
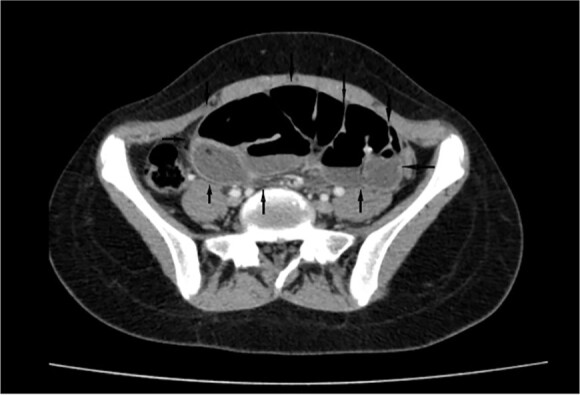
Retrospective review of ECT (axial section), a membrane encapsulates the small intestine centrally indicates the diagnosis of ACS (black arrows).

**Figure 5:**
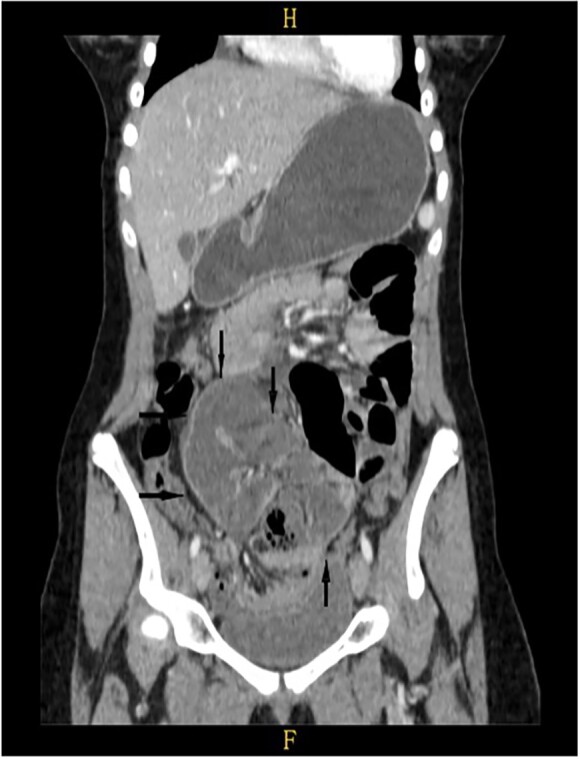
Retrospective review of ECT (coronal section), a membrane encapsulates the small intestine centrally indicates the diagnosis of ACS (black arrows).

On a retrospective review of our patient’s ECT, we recognised a membrane encapsulating the small intestine centrally, which indicates the diagnosis of ACS (Figs 4 and 5).

The specific ECT findings in patients having recurrent attacks of subacute SBO with unknown causes help in diagnosing and determining the proper management of ACS.

However, it is acknowledged that we misdiagnosed ACS as acute appendicitis that provoked the surgery.

Surgery is the traditional therapy for ACS as most of the cases are discovered post-operatively**.** In mild attacks of SBO with the specific ECT findings, conservative management should be kept in mind.

Conservative management is still the treatment of choice for managing adhesive SBO with high success rates. In non-responding cases, surgical dense adhesiolysis might by followed with recurrent severe adhesions [6]. This fact supports the consideration of conservative treatment as the first line of therapy for mildly symptomatic ACS.

Conservative treatment consists of observation alone or insertion of a long nasointestinal decompression tube with medical treatment. Some reports have demonstrated successful trials by using steroids, colchicine, tamoxifen or azathioprine [7].

The incidence rate of ischemia and perforation is rare in ACS. The thickened fibrotic membrane encapsulating the bowel with the dense adhesions prevent the vascular pedicle from twisting [8]. Therefore, the necessity for bowel resection in such a case is less common compared with when a band is the cause of intestinal obstruction [6].

On comparison with conservative treatment, surgery has a high incidence of iatrogenic complications (fistula formation, abscess with sepsis and recurrent adhesions) when attempting lysis for the severe adhesions between bowel loops [8]. In the largest study done in China including 82 ACS patients, 19 patients developed post-operative bowel obstruction, who were treated conservatively, 6 patients complicated with intestinal fistula and 3 of them died [9].

Most of the patients with ACS have intermittent mild attacks of SBO and rarely develop peritonitis or strangulation. When ACS is diagnosed with ECT, the treatment for such a case is a balance between conservative and surgical treatment. We believe that surgical management can be preserved for the cases with peritonitis or when not responding to the conservative management.

Notably, the limited number of reported cases was of patients who have sought a medical consultation and, maybe, there are more patients still not diagnosed because they have mild or no symptoms. This might be supporting the conservative management as the primary choice for mildly symptomatic ACS. More comprehensive studies are necessary to establish the proper management for ACS. Increasing the published reports will improve the awareness of the specific radiological findings and avoid the patient unnecessary surgery.
